# Estimates of Abundance and Trend of Chilean Blue Whales off Isla de Chiloé, Chile

**DOI:** 10.1371/journal.pone.0168646

**Published:** 2017-01-12

**Authors:** Barbara Galletti Vernazzani, Jennifer A. Jackson, Elsa Cabrera, Carole A. Carlson, Robert L. Brownell

**Affiliations:** 1 Centro de Conservación Cetacea—Casilla 19178 Correo 19, Santiago, Chile; 2 British Antarctic Survey, High Cross, Madingley Road, Cambridge, United Kingdom; 3 College of the Atlantic, Bar Harbor, ME, United States of America; 4 Southwest Fisheries Science Center, NOAA, Monterey, California, United States of America; Northwestern University, UNITED STATES

## Abstract

Since 1970, blue whales (*Balaenoptera musculus*) have been seen feeding in the waters off southern Chile during the summer and autumn (December to May). Investigation of the genetic, acoustic and morphological characteristics of these blue whales shows that they are a distinct but unnamed subspecies, called the Chilean blue whales. Photo-identification surveys have been conducted in the waters off northwestern Isla Grande de Chiloé, southern Chile from 2004–2012 and Isla Chañaral, central Chile in 2012. Over this time, 1,070 blue whales were encountered yielding, after photo-quality control, 318 and 267 unique photographs of the left and right side of the flank respectively. Using mark-recapture analysis of left and right side photographs collected from Isla Grande de Chiloé (2004–2012), open population models estimate that ~570–760 whales are feeding seasonally in this region. POPAN superpopulation abundance estimates for the same feeding ground in 2012 are 762 (95% confidence intervals, CI = 638–933) and 570 (95% CI 475–705) for left and right side datasets respectively, very similar to results from closed population models. Estimates of trend revealed strong variation in abundance, peaking in 2009 and [suggesting] fluctuating use in the survey area over time, likely related to the density of their prey. High inter-annual return rates suggest a degree of site-fidelity of individuals to Isla Grande de Chiloé and that the number of whales using this feeding ground is relatively small.

## Introduction

Three subspecies of blue whales are currently recognized in the Southern Hemisphere: the pygmy blue whale (*Balaenoptera musculus brevicauda)* in the sub-Antarctic zone; the Antarctic blue whale (*B*. *m*. *intermedia*) which summers in the Antarctic Zone [1], and an unnamed subspecies, the Chilean blue whale off Chile, which is intermediate in size between pygmy blue whales and Antarctic blue whales [2]. This unnamed subspecies has been accepted by the Taxonomy Committee of the Society for Marine Mammalogy [3], considering geographic, acoustic and genetic evidence that Chilean blue whales are significantly differentiated from Antarctic blue whales [4–6] and that Chilean blue whales are as different from Antarctic blue whales as they are from the pygmy blue whale population from Australian waters [4].

**Fig 1:**
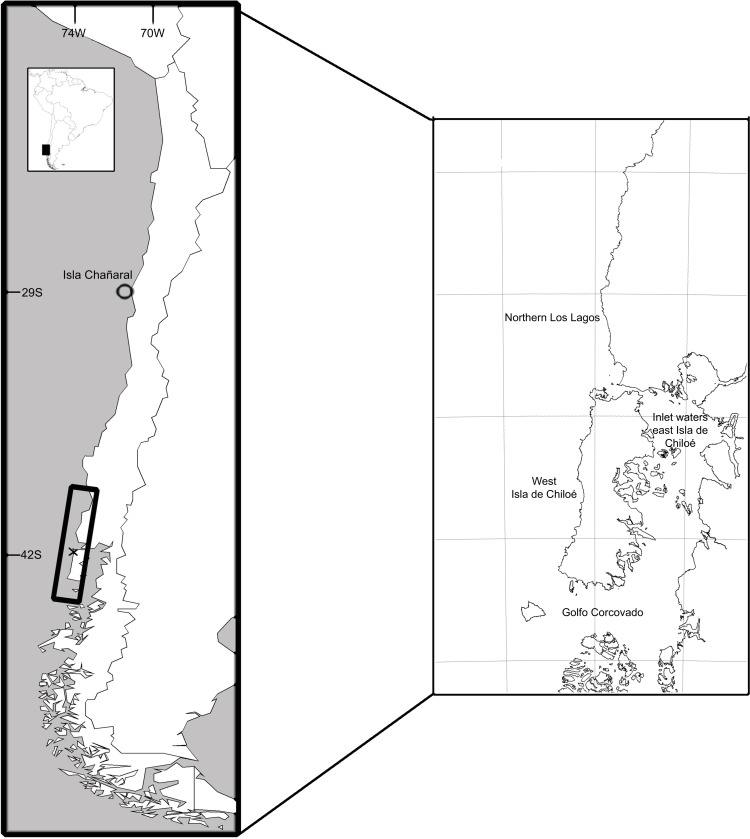
Blue whale study areas in central and southern Chile. Circle: waters around Isla Chañaral, northern Chile; Rectangle: southern Chile survey area in the region of Isla Grande de Chiloé.

Blue whales were targeted worldwide by 20^th^ century modern whaling, with an estimated 5,782 caught by whaling stations and floating factories working offshore of Peru, Ecuador and Chile from 1908 to 1971 [7]. However, it is unclear how many blue whale populations were actually hunted along the west coast of South America. Since 1970, blue whales have often been seen feeding during the austral summer and autumn (late December to early May) in southern Chilean waters spanning the northern Los Lagos region (41°S), south to the outer coast of Isla Grande de Chiloé (Chiloé), south to Isla Guafo (43.6°S) and eastward into the Golfo Corcovado around the northern islands of the Chonos Archipelago (Fig 1) [8–14]. Recently, additional sightings have been reported during autumn and early winter in the inlet waters east of Chiloé near the mainland [15, 16]. In central Chile, an additional feeding aggregation of blue whales off Isla Chañaral, was reported in 2012 [17] and blue whales also used this area in the 1997/1998 season during the major El Niño event [14].

Based on surveys conducted from the IWC-SOWER 1997/98 blue whale cruise off central Chile [11], Williams *et al*. [7] used spatial modeling methods to obtain an abundance estimate of 303 (95% CI: 176–625) whales. This estimate represents the number of whales present in the sampled area (Fig 1) in December during their southward migration, and does not represent the total abundance of Chilean blue whales nor the abundance of the whole southern Chile feeding ground, especially given that 363 individual blue whales were photo-identified during 2004–2010 off Chiloé, southern Chile [14].

Capture-recapture techniques using photo-identified individuals have been increasingly used to estimate the population size of large whales, including blue whales in the eastern North Pacific and western Australia [18–21]. These approaches have the advantage over line transect methods that they can go beyond the number of animals using an area to investigate both the population composition and patterns of usage of an area over time [22, 23]. Blue whales are individually identifiable from the unique pattern of mottling on both sides of the body with the dorsal fin as a reference point [24], unique patterns on their flukes [25], and in some cases permanent scars can be used to identify or confirm individuals [14]. Centro de Conservacion Cetacea (CCC) has been conducting the Alfaguara (blue whale) Project since 2004. Individual photo-identifications of blue whales have been systematically collected using photographs of left and right sides of each whale and then compiled into a photographic catalogue [14].

Here we measure the inter-annual changes in abundance of blue whales feeding in the waters off Chiloé, using mark-recapture models and photo-identification data obtained from nine years of surveys by the Alfaguara Project off Chiloé in southern Chile from 2004 to 2012 and off Isla Chañaral in northern Chile during 2012.

## Materials and Methods

No permits were required for the collection of data used in this study.

### Study area

The primary/main survey area was off northwestern Chiloé, between Chacao Channel (41°45’S) and south of Isla Metalqui (42°12’S) within 12nm from the coastline, on board the 7m *Alfaguara* research vessel (Fig 1). One marine survey was conducted off northern Los Lagos in 2008 and one around the Golfo Corcovado in 2004 on board a 30m Chilean Navy surveillance vessel [14]. Surveys were generally conducted from end January to late April (S1 Table). In 2012, Isla Chañaral, located at 29°S, 73°W in northern Chile also was surveyed on board a small tourist boat from 24–27 February [17].

Data collected during marine surveys included photo-identification, group composition, behavior, weather and sea conditions, associated fauna and sea surface temperatures (SST). The position of a whale or group of whales was determined using GPS.

### Catalogue compilation and photo-quality control

Each season, photo-quality is assessed for every blue whale photo-identification. Photographs which are of poor resolution or have a bad image angle are not retained. Each photograph is assessed for quality in terms of contrast, angle and focus. Photographs are then contributed to the master CCC catalogue, which consists of separate photographic collections for the left and right sides of the head region, dorsal fin, flank and caudal peduncles. The catalogue contains “medium” and high quality quality pictures as lower quality images can be useful for discerning whale movements with photo-identification, but are not necessarily of sufficient quality for mark-recapture purposes.

For mark-recapture analyses, photographs must be good quality to enable a high probability of matching them with future photographic sightings of the same whale [20]. The evaluations of photographic quality and individual distinctiveness are subjective judgments and therefore the selection of data sets to be used in mark-recapture analysis is also subjective. Friday *et al*. [26, 27] evaluated photographic quality and individual distinctiveness as well as their effect on capture-recapture estimates using North Atlantic humpback whale photo-identifications. They found that most judges can agree when evaluating photographic quality and individual distinctiveness and that an appropriate balance between precision and bias in abundance estimates was achieved by removing the lowest-quality photographs.

For mark-recapture purposes we conducted an in-depth photo quality control assessment, adapting the method used by Friday *et al*. [26, 27] to our case of blue whales off Chile. Two judges scored the left and right side datasets independently and two methods were explored. Each judge has ten years of experience with blue whale photographic identification. Photographic quality was scored using three specific variables (contrast, angle and focus) and one general variable (overall quality). The first method considers overall quality only and the second method uses an average of specific variables to predict photographic quality. The judge was asked to score each variable on a scale of four, using the quality criteria above from the best photographs to the worst (3 = very good, 2 = good, 1 = acceptable, and 0 = unacceptable). The judge was also asked to accept or reject a photograph. If any of the specific variables were unacceptable, the photograph was discarded.

Therefore, several datasets were constructed for each judge and method, for left side and right side photographs of blue whales, collected during surveys spanning 2004–2012.

Individual histories rejected for mark-recapture analyses varied between methods and judges, from 30% to 64%. The judge who provided the more rigorous evaluation was chosen, on the basis that this was less likely to positively bias abundance estimates with false negatives. The method based on scoring specific photo-quality variables was chosen for similar reasons. Individuals with a photograph quality score of 1.3 or higher were retained in the dataset; this score was chosen in order to include all photographs that were above ‘adequate’ quality for mark-recapture analysis.

### Photo-identification matching

Photographs of individual blue whales were compared within season to determine the number of individuals sighted and resight matches. All individual whales then were compared to the master CCC catalogue to determine if they were new or known individuals. Overall consistency in research design, data collection techniques and data analysis allowed for between-year comparisons [14, 28].

### Mark-recapture analysis

As only one right side photo-identification was obtained in 2004, we removed this year from mark recapture analysis of the right side dataset. In order not to violate assumptions of survey area comparability through time, we measured mark resight abundance within the Chiloé dataset only and did not include sightings from Isla Chañaral. Primary analyses were therefore conducted on left side data from Chiloé from 2004–2012 and right side data from 2005–2012. To check the sensitivity of these data to small sample sizes collected during 2004, 2005 and 2012 (S1 Table) we also analysed a shorter dataset spanning 2006–2011 for comparative purposes.

To determine the goodness-of-fit of the data to standard Cormack-Jolly-Seber models, we tested the goodness-of-fit of these data using single-state tests 3.SR, 3.SM, 2.CT and 2.CL in the program U-CARE V2.3.2 [29], which tests for transients (individuals with unequal resight probabilities) and trap dependence (i.e., initial sightings of some whales are followed by lower or higher than expected probability of resights), recognizing that any evidence for trap dependence would most likely be a sign that some other effect was at play.

### Mark-recapture analysis of abundance

Closed and open population models were explored on the basis that closed models can better account for capture heterogeneity within the data, but the long time period of the survey make open population models (which include a emigration/mortality component) more appropriate for these data.

Closed populations models were investigated using CAPTURE software. The fit of alternative models of capture heterogeneity was also investigated and models were discriminated using a model selection algorithm developed by Otis et al. [30].

For open population abundance estimation we chose the POPAN model implemented in MARK [31], an extension of the Jolly-Seber model which assumes that whales encountered over the survey period are a component of a larger ‘superpopulation’ using Chilean coastal waters to feed. The superpopulation size is interpreted as the total number of animals ever present during the study period and does not represent the number present at any particular point in time.

POPAN models can be used to calculate apparent survival (φ), probability of capture (p), probability of entry of members of the superpopulation (i.e. through birth or immigration) into the population (β) and the total superpopulation size (N_P_). Since a number of parameters are unidentifiable when using the fully time (t) dependent POPAN model (φ_t_ p_t_ β_t_) we only explored POPAN models with constant apparent survival. When capture probabilities were not fully identifiable, we constrained two or more capture probabilities to be equal (e.g. p_1_ = p_2_, p_1_ = p_8_), with the constrained set chosen based on the Akaike Information Criterion (AIC) goodness-of-fit of the constraint models relative to free parameter model. Models were fitted using a logit link function for survival φ and capture probabilities p, a log link for N_p_, and the multinomial logit link function to constrain entry probabilities β to sum to ≤ 1 for the POPAN model.

The Pradel model [32] was used to estimate abundance trends. This model can be used to estimate realized growth rates from the population (λ) as well as apparent survival (φ) and probability of capture (p_P_). As with the POPAN model, all Pradel models were constrained to have constant apparent survival through time. Estimates of annual abundance and their associated confidence intervals were derived from the capture probabilities of the best-fitting model, by dividing the numbers of captures in each season by their associated probabilities. For both POPAN and Pradel models, the best fitting models were determined according to AIC scores of goodness-of-fit.

## Results

During 109 marine surveys totaling 591 hr conducted off Chiloé from February to April 2004–2012, 710 groups of blue whales containing a total of 1,070 individuals were encountered. In February 2012, during four marine surveys totaling 26 hr conducted off Isla de Chañaral, 17 groups of 22 blue whales were encountered (S1 Table). Sightings per hour off Isla de Chiloé ranged from <1/hour (2004 and 2012) to 10-20/hr (2005, 2008 and 2010, S1 Table). A total of 406 individual blue whales were photo-identified from the left side and 419 from the right side. Multiple-year resights of 101 and 95 individuals were observed for left and right side photographs respectively, including 21 left and 19 right sides sighted over three years and 4 individuals sighted in four years (Table 1). During the 2012 field season, no matches were found between the individuals off Isla de Chañaral and those catalogued off Chiloé. Fisher’s exact test comparing observed versus expected resights rejected the null hypothesis that resight rates were evenly distributed among the photo-identifications collected off Chañaral and Chiloé at p<0.05 for both the left and right side datasets.

**Table 1:** Chilean pygmy blue whales identified by photographs, shown by year of capture and recapture.

	Year									
Left side	2004	2005	2006	2007	2008	2009	2010	2011	2012	2012
									Chiloé	Chañaral
Ind captured	4	11	44	70	85	50	82	52	6	14
Cumulative ind. captured	4	15	58	120	177	217	274	302	304	318
% resights		0	2.3	11.4	36.5	24	42.7	65.4	67	0
	Year of recapture									
Initial capture year	2004	2005	2006	2007	2008	2009	2010	2011	2012	2012
2004		0	0	1	1	0	0	0	0	0
2005			1	1	3	0	1	1	0	0
2006				6	9	4	7	1	0	0
2007					18	3	8	7	1	0
2008						5	11	12	1	0
2009							8	7	2	0
2010								6	0	0
2011									0	0
	Year									
Right side	2004	2005	2006	2007	2008	2009	2010	2011	2012	2012
									Chiloé	Chañaral
Ind captured	1	8	54	61	92	39	60	33	7	8
Cumulative ind. captured	1	9	62	116	174	203	242	255	259	267
% resights		0	1.9	11.5	41.3	33.3	51.7	81.8	42.9	0
		Year of recapture								
Initial capture year	2004	2005	2006	2007	2008	2009	2010	2011	2012	2012
2004		0	0	0	1	0	0	0	0	0
2005			1	1	3	0	0	1	0	0
2006				6	13	4	7	1	0	0
2007					21	4	6	8	0	0
2008						5	11	9	1	0
2009							7	4	2	0
2010								4	0	0
2011									0	0

After photo-quality control and dataset selection, 22% of catalogued individuals were discarded from the left side and 36% from the right side. One of the individuals removed was observed in four different years, four in three years, 19 and 15 seen twice for left and right side respectively and 64 and 132 seen only once for left and right side respectively. Sighting histories from 318 individuals with left side photographs and 267 with right side photographs were used to perform the analyses. Cumulative resights through time for 2004–2012 off Chiloé are plotted in S1 Fig.

### Estimates of abundance and trend

Goodness-of-fit tests revealed a significant transience signal in the left side dataset (p<0.05) with a two-sided test and a one-sided test, and with standardized log-odds ratios (S2 Table). This was not significant for the right side dataset. Annual transience estimates reveal that the significant signal comes from 2009, with significantly fewer 2009 whales photo-identified in the following years than expected for the left side dataset. A significant result was also found for test 2.CL for the right side dataset in 2007, rejecting the null hypothesis that whales sighted in 2008 had similar probabilities of being sighted and not sighted two years prior in 2006.

Model selection of closed models in CAPTURE supported time-varying models for both left and right side photographs, with capture heterogeneity models most strongly supported for both left and right datasets (S3 Table). The Mth Chao model yielded an overall abundance estimate of 741 (95% CI = 607–937) and 542 (95% CI = 447–685) for left and right side datasets respectively over 2004–2012.

The 2004–2012 POPAN and Pradel models explored in this analysis are shown in S4 and S5 Tables, sorted by AIC scores. Only models with constant survival and fully identifiable capture probabilities were compared. The best fitting POPAN models were ϕ_(.)_ p_(1 = 2 = 9,3 = 7,t)_ PENT_(1 = 5,2 = 8,t)_ N_(.)_ and φ _(.)_ p_(1 = 8,2 = 6,5 = 7,t)_ PENT_(t)_ N_(.)_ respectively for left and right side photographs, though alternate models provided very similar fit (S4 Table). Estimates of apparent survival and superpopulation abundance were congruent between the two datasets. Model averaged superpopulation abundance estimates were N = 761 (95% CI = 614–908) for left side and N = 569 (95% CI = 455–683) for right side photographs respectively (Table 2), while apparent survival was estimated at φ = 0.88 (SE 0.04) and φ = 0.91 (SE 0.04) for left and right side photographs respectively.

**Table 2:** Chilean blue whale apparent survival (ϕ), superpopulation size ($\hat{N}$) and annual population abundance (*N*_*t*_) estimates for 2004–2011 using the POPAN population model.

	Left side			Right side		
	ϕ(.) p(1 = 2 = 9,3 = 7,t),PENT(1 = 5,2 = 8,t)			ϕ(.) p(1 = 8,2 = 6,5 = 7,3,4),PENT(t)		
ϕ	0.88 (0.88)			0.91 (0.91)		
SE	0.04 (0.04)			0.04 (0.04)		
$\hat{N}$	762 (761)			570 (569)		
SE	74 (75)			58 (58)		
CI	638–933 (614–908)			475–705 (455–683)		
Year	p_t_	N_t_	CI	p_t_	N_t_	CI
2004	0.02	195 (187)	93–408 (16–357)			
2005	0.02	456 (456)	373–557 (360–552)	0.02	389 (381)	315–481 (246–515)
2006	0.11	402 (402)	327–494 (317–488)	0.15	353 (353)	287–435 (279–427)
2007	0.20	355 (356)	279–453 (268–444)	0.19	320 (320)	252–407 (243–397)
2008	0.27	314 (314)	232–424 (218–410)	0.31	291 (291)	217–390 (204–533)
2009	0.09	561 (561)	468–672 (457–664)	0.09	444 (444)	365–540 (355–533)
2010	0.17	495 (496)	397–618 (382–609)	0.15	403 (403)	316–513 (302–504)
2011	0.11	437 (438)	330–581 (309–567)	0.09	366 (366)	269–497 (249–483)
2012	0.02	386 (387)	271–551 (158–616)	0.02	332 (332)	227–485 (197–467)

Shown in parentheses are the estimates derived from model averaging over all POPAN mark-recapture models explored in MARK.

All Pradel models with constant apparent growth (λ) were more poorly fitting to the data than models with variable growth (AIC differences were >8 in all cases, S5 Table). Apparent survival estimates were very similar between left and right datasets and slightly higher than those derived from the POPAN model, with φ = 0.92 (SE 0.06) and φ = 0.91 (SE 0.05) for left and right sides respectively. Estimates of capture probability were also consistent across left and right side datasets (Table 3), although estimates of λ were more dissimilar between datasets, with λ<1 found in 2008, 2010 and 2011 and λ>1 found in 2009. Years 2006, 2007 and 2012 showed contrasts between the two datasets in terms of estimated growth or decline, probably influenced by low sample sizes in the years 2004–2005 and 2012. When λ was constrained to be constant, estimated apparent growth rates were very similar for left and right sides: λ = 1.03 (95% CI = 0.97–1.10) and λ = 1.02 (95% CI = 0.95–1.08) respectively. There are broad confidence intervals on both estimates which do not exclude the possibility of a constant-size population, slight population growth or a slow decline over the survey period.

**Table 3:** Chilean blue whale apparent survival (ϕ) and apparent population growth (λ) for 2004–2012 using the PRADEL population model.

	Left side			Right side		
	ϕ(.) p(1 = 2 = 9,3 = 6,4 = 7,5,8) λ(1 = 5,3 = 6 = 7 = 8,2,4)			ϕ(.) p(1 = 8,2 = 3 = 6 = 7,4,5) λ(1 = 5,3 = 6,2 = 7,4,8)		
ϕ	0.92			0.91		
SE (CI)	0.06 (0.71–0.98)			0.05 (0.73–0.98)		
λ (constrained to λ(.))	1.03			1.02		
SE (CI)	0.03 (0.97–1.10)			0.03 (0.95–1.08)		
Year	p_t_	λ	N_t_	p_t_	λ	N_t_
2004	0.02		234 (90–623)			
2005	0.02	2.36	644 (247–1714)	0.02		419 (227–778)
2006	0.07	1.02	643 (410–1023)	0.13	0.91	432 (303–627)
2007	0.14	0.78	491 (340–727)	0.13	1.17	488 (342–709)
2008	0.27	0.64	314 (223–465)	0.33	0.58	281 (206–407)
2009	0.07	2.36	731 (466–1162)	0.07	1.91	537 (300–991)
2010	0.14	0.78	576 (399–852)	0.13	0.91	480 (336–697)
2011	0.12	0.78	447 (280–739)	0.13	0.58	264 (185–383)
2012	0.02	0.78	351 (135–935)	0.02	1.17	785 (426–1459)

**Fig 2:**
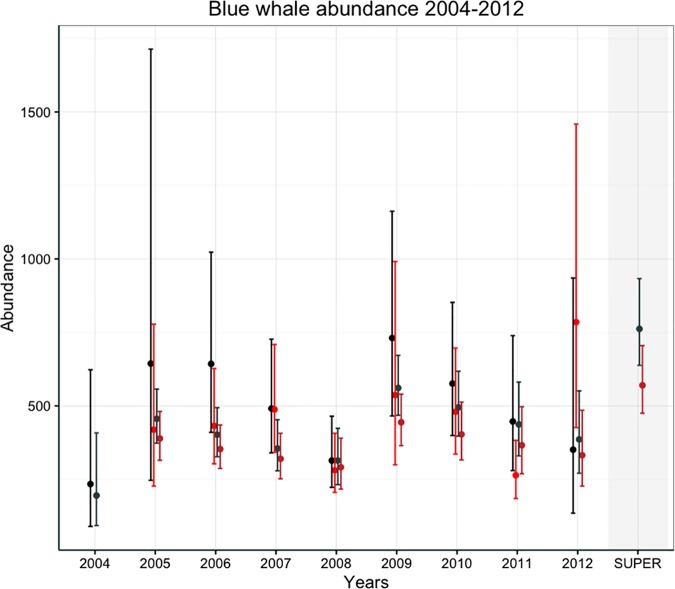
Estimates of Chilean blue whale annual abundance from 2004–2012 and POPAN superpopulation abundance (measured from January/February in each year). Estimates and 95% confidence intervals are, from left to right, Pradel left sides (red), Pradel right sides (black), POPAN left sides (red), POPAN right sides (black), with POPAN superpopulation estimates for POPAN left and right sides respectively.

Annual abundance estimates derived from the best fitting POPAN and Pradel models are shown in Fig 2 and reveal a fluctuating pattern. Data reveal a significant shift in habitat use between 2008 and 2009, showing a significant influx of whales (a >200% increase from 2008) into this feeding ground during the 2009 season.

Capture probabilities estimated from both left and right datasets were over 10% in all years but 2009 (Tables 2 and 3). Considered together with resight rates of over 60% in 2011, these data reveal high inter-annual fidelity of blue whales to waters off Chiloé. However inter-annual fluctuations (Fig 2) and low apparent survival estimates (Tables 2 and 3) suggest use of this feeding ground varies between years. It is likely that factors related to annual habitat quality influence the observed annual abundances.

## Discussion

### Blue whale abundance and fidelity to Isla de Chiloé

Blue whales feeding in the waters of our survey area off Chiloé are estimated to number ~570–760 whales, with periodic fluctuations in abundance suggesting that use of this area varies between years. An earlier survey offshore the central Chilean coast was conducted during the migration season north of Chiloé (18–38°S) in December 1997, with ~303 whales estimated [7]. The offshore survey in 1997 covered part of the migration corridor and the surveys off Chiloé only covered part of the feeding ground[s]. The abundance estimates obtained from these two surveys are not comparable, since they were made in different places, using different survey techniques, with the earlier survey conducted in December whereas the mark-recapture surveys were conducted between January and April off Chiloé over a number of years. All of the blue whales observed offshore central Chile in December 1997 were assumed to be migrating south to their feeding ground[s].

Other blue whales assumed to also be of the Chilean pygmy type are most abundant during the austral summer north of 09°S, in the waters within 60 nm off Peru [33]. The peak abundance of blue whales off Peru is January and February, concurrent with the Chiloé whales, suggesting that the Peruvian whales belong to one or more different populations [33].

Hucke-Gaete [34] reported attaching satellite tags to blue whales south of Chiloé in the Golfo Corcovado. One whale migrated along Nazca Ridge (ca. 1300 km offshore) to around latitude 23° S before the tag failed. Another blue whale was photographed and biopsied west of the Galapagos Islands (01°52’S) on 6 November 1998 and matched genetically and photographically with a whale sampled south of Chiloé (43°52’S) on 23 February 2006 [35]. These observations strongly suggest that at least some Chilean blue whales from southern Chile feeding grounds migrate to west of the Galapagos Islands and into the Eastern Tropical Pacific (ETP) for breeding and calving, as hypothesized by Reilly and Thayer [36].

### Feeding ground site fidelity but no population structure?

While summer sightings of blue whales are relatively continuous along a small section of the Chilean coast, the high resight rate suggests whales have high inter-annual fidelity to the Chiloé region for feeding. There were 22 photo-identifications made in the north around Isla Chañaral in February 2012 but no matches to any whales previously identified in Chiloé (although a resighting between these areas has been reported previously, [14]). Of all whales sighted from 2004 to 2012 off Chiloé, 41 and 46% were between–year resights (for left and right sides respectively). Among whales sighted off Chiloé in 2012, 43% of left sides were resighted and 66% of right sides. If Isla Chañaral was equally connected to Chiloé, ~6 left side resights would be expected from the 2012 sightings and ~5 right side resights, but none were found.

The possibility of multiple distinct inter-annual feeding grounds is also supported by the pattern of past catches: following four decades of whaling along the Chilean coast, Clarke [37] reported unexploited offshore feeding grounds at 30–35°S in the Humboldt Current; multiple fin whale sightings were made in this vicinity during October/November 1958. Subsequently Aguayo [38] reported that land stations in this region between 1964 and 1967 took close to 500 fin whales and 700 blue whales, with 378 blue whales killed in a single season [39]. It should be noted that all photo-identification surveys have been conducted between late January and April, and migratory movements of whales through feeding areas (e.g. up the coast or offshore to lower latitude wintering grounds) may mean that different connectivity patterns between Chiloé and Isla Chañaral might be observed if surveys were conducted outside the summer season.

Regional feeding ground fidelity might be indicated by these mark-recapture results, implying a degree of long-term seasonal segregation of whales. However the apparent lack of genetic substructure observed along the Chilean coast [5] suggests seasonal segregation is limited. Other whale species with common wintering grounds but long-term fidelity to different feeding areas in the North Pacific exhibit female-mediated population structure (e.g. humpback and gray whales, [40, 41]), but arguably these feeding areas are spread over greater geographic distances than seen here, and have geographically distinct migratory routes. Even without genetic divergence between geographic sites, differences in the frequencies of maternally inherited haplotypes between sites can be expected due to the long-term, maternally influenced fidelities of females to particular feeding areas. Genetic structuring can also occur due to assortative mating on the migratory route before arrival on the breeding grounds. Photo-identification catalogue comparisons between blue whales feeding in summer in the Golfo Corcovado (south of Chiloé, Fig 1) and blue whales wintering in the ETP have revealed a direct connection between southern Chile feeding grounds and the Galapagos Archipelago [35]. If Chilean blue whales use a common migratory route to northerly breeding grounds from multiple feeding areas, this could reduce or even eliminate assortative mating amongst feeding ground members en route. Available data from satellite tags and sighting surveys suggest that this migratory route is likely offshore [11, 34, 35]. The ETP is a complex region for baleen whales, as some blue whales satellite-tagged off southern California migrate to the ETP [42], and some humpback whales from the Southern Hemisphere also use the ETP [43].

Alternatively, there could have been population sub-structuring in the past, eliminated by recent whaling so that the current pattern reflects a very recent expansion of a small remnant population of whales into former feeding ground habitat following whaling. Torres-Florez et al. [44] also noted haplotype diversity within the species is higher than might be expected considering the recent whaling impact, and hypothesized that this might be due to some degree of past sub-structuring. They suggest that the relatively high diversity seen in the Chilean blue whales (0.890) relative to pygmy blue whales [45] off Australia may be due to long-term feeding ground site fidelity along the coast (as suggested by the present study) despite the lack of evidence for genetic sub-structure. Torres-Florez et al. [44] did not find evidence for a recent population bottleneck due to whaling, but noted that the genetic bottleneck signal may be lagged due to the long generation times within this species and relatively short time since whaling. If the population is now expanding from a bottleneck, regional fidelity, while strong, may have been in effect only in the last 2–3 generations (assuming a generation time of about 30-years) in newly colonized areas. Genetic structure over such a short period would not be easily detectable, both because of the short timeframe of expansion, and because regional genetic diversity is relatively low, so the ability to resolve significant frequency differences between feeding sites is also reduced.

A final possibility, and the one we believe is most likely, is that Chilean blue whales regularly visit multiple feeding sites along the Chilean coast during the summer season, depending on annual oceanographic conditions affecting krill density. This would explain both the regional site fidelity and the lack of population structuring between feeding sites. Transient use of the Chiloé feeding ground by some individuals is suggested by left side dataset goodness-of-fit tests (S1 Table), yet the pattern of resights also suggests high inter-annual fidelity to Chiloé by a sizeable proportion of whales. Analysis of the relative proportions of resident and transient whales within this data for 2006–2011 indicates that 40–45% are resident [46], so roughly even proportions of sighted whales are resident and transient, consistent with behavioural site fidelity but also long-distance movements during the feeding season. Satellite telemetry indicates that individual North Pacific blue whales travel long distances along the US west coast (predominately the coast of California) each season, feeding on and transiting between large prey patches [47]. Chilean blue whales may range over similar areas to feed. Such widespread movements do not quite marry with the apparently distinct groups of whales seen off Chiloé and Isla Chañaral (i.e., no resights) but it must be cautioned that surveys were conducted at Isla Chañaral during a season where a significant shift in blue whale distribution was also observed; further evidence is therefore required to clearly establish whether populations differ between these two areas. Further dedicated coastal sightings surveys will be required to investigate overall Chilean blue whale population and connectivity and understand the drivers of these apparently contradictory patterns.

### Population trends and oceanographic context

Following exploitation, a number of baleen whale populations worldwide have shown increasing trends in abundance [48–54]. Best [52] reported that over 80% of baleen whale populations that have been monitored show evidence of population increase. Even some of the smallest baleen whale populations show increases when monitored, like the western gray whale [55]. Thus following past exploitation, Chilean blue whales are also likely to be increasing in abundance, but the survey data from Chiloé reveal fluctuations in abundance which are not recruitment-driven. When models with a single population growth rate are fitted, these suggest that the rate of population growth is very low, but with broad confidence intervals including both positive and negative trends in abundance. In this regard, it is important to note that increasing threats have been reported for this austral feeding ground[s], including a dead blue whale from ship strike [56], highlighting the need of further attention for this population and increase in conservation efforts.

The path of the Antarctic Circumpolar Current (ACC) is through the Drake Passage, between Cape Horn and the Antarctic Peninsula. The north-flowing Cape Horn Current (CHC) is caused by northward movement of part of the ACC along the coast once it reaches Tierra del Fuego. The Humboldt Current (HC) starts along the northern limit of the CHC and flows north to Peru and is considered to be the world’s most productive marine ecosystem and the largest upwelling system. This is therefore a region of significant deep-water upwelling and primary productivity, stimulating large seasonal phytoplankton blooms resulting in high densities of various krill species resulting in multiple feeding sites. Water masses in the Chiloé region are also fed by river run-off, glacial ice-melt and tidal currents that enhance upwelling. In addition, there are very strong tidal currents between the mainland and Chiloé in the Chacao Channel that enhance upwelling. However, in recent years the rich productivity of the HC has been frequently perturbed by reoccurring El Niño Events.

Assessment of recovery from whaling to date suggests the Chilean blue whale population was no lower than 7% of pre-exploitation numbers in 1998 [7], but would now be at higher levels given the larger abundance estimates in this paper. Mark-recapture models fitted with a time-constant population growth parameter were poorly fitting to the dataset, and yielded annual growth estimates of 2–3%, with wide confidence intervals including negative values (i.e., population decline). However, a period of significant flux was detected in 2008 and 2009. Since trends are likely primarily driven by habitat use rather than population recruitment, these results suggest differing use of the feeding ground over the two years. In 2008 and 2011, over 35 and 65% of photo-identifications matched back to previous sightings; in the context of the nine-year period, these resight rates were unusually high (S1 Fig). Fewer whales were photo-identified in 2009 (n = 124) and resight rates for these whales dipped by ~8%, suggesting possibly an influx of different whales over the summer survey period. Low sighting rates (per hour of observation) off Chiloé in 2012 may have been due to a shift in blue whale distribution about 1,445 km to the north (off Isla Chañaral) in that year [17].

In the North Atlantic, sightings surveys spanning 13 years suggested North Atlantic blue whale abundance increased at 9% per annum on feeding grounds west of Iceland from 1987–2001, although with broad confidence intervals spanning 1–17%, and as with the current survey, it is not known how representative of the population distribution this survey area was [54]. Similarly, sightings rates varied significantly over time between different parts of the survey area, suggesting fluctuation in use of the feeding ground. In the eastern North Pacific blue whales feed further north during years when the environment is perturbed (for example during El Niño periods), rather than feeding on prey patches of reduced size on their regular feeding grounds [47, 57]. The warm phase of the Southern Oscillation (El Niño) has particularly pronounced effects along the west coast of South America, as it brings an influx of nutrient-poor equatorial water into the Humboldt Current, increasing water temperatures and reducing upwelling along the coast, with impacts on local fisheries (e.g. [58, 59]). El Niño effects are therefore likely to influence Chilean blue whale distribution and habitat use between seasons. With continued surveys in this region, capture probabilities measured from mark-recapture data can be compared with environmental time series (e.g. chlorophyll and SST variation) to investigate these relationships in more detail.

## Conclusions

Our nine years of data reveal that the Chiloé feeding ground is used by a small number of whales in the mid-hundreds which have high inter-annual fidelity to this region. The data did not detect a significant trend in abundance over this timeframe, with changing abundance in this region more likely reflecting seasonal differences in habitat use than the lack of population recruitment.

## Supporting Information

S1 Fig Cumulative resightings of Chilean blue whales off Isla de Chiloé from 2004–2011. (PDF)

S1 Table Summary of Chilean blue whale sightings, sightings per unit effort (SPUE) and photo-identification after photo-quality control. (DOCX)

S2 Table Summary of Chilean blue whale results from U-CARE tests of goodness-of-fit between the left and right side datasets and various Cormack-Jolly-Seber models. (DOCX)

S3 Table Chilean blue whale abundance estimates from closed mark-recapture models calculated in CAPTURE for 2004/5-2012. (DOCX)

S4 Table Summary of top p/ϕ-identifiable POPAN models explored in MARK for Chilean blue whales. (DOCX)

S5 Table Summary of top identifiable Pradel models explored in MARK for Chilean blue whales. (DOCX)

S1 Data Left side mark resights of Chilean blue whales from Isla de Chiloé 2004–2012. (INP)

S2 Data Right side mark resights of Chilean blue whales from Isla de Chiloé 2005–2012. (INP)
