# Supplementary material for: Estimates of Abundance and Trend of Chilean Blue Whales off Isla de Chiloé, Chile
Source: PLoS One. 2017 Jan 12;12(1):e0168646. doi: 10.1371/journal.pone.0168646 (PMC5231374; doi:10.1371/journal.pone.0168646)
Supplement: S1 Table — (DOCX) [file pone.0168646.s002.docx]

**Table S1 –** Summary of sightings, sightings per unit effort (SPUE) and photo-identification after photo-quality control

| **Year** | **Sampling Period (mo/d)** | **# surveys** | **Hours of obs** | **Groups of blue whales encountered** | **# blue whales encountered** | **SPUE** | **Left side photo-ID after quality control** | | **Right side photo-ID after quality control** | |
| --- | --- | --- | --- | --- | --- | --- | --- | --- | --- | --- |
|  |  |  |  |  |  |  | New individuals | Individuals sighted previously | New individuals | Individuals sighted previously |
| 2004 | 02/25 – 03/15 | 2 | 17:35 | 2 | 4 | 0.23 | 4 | 0 | 1 | 0 |
| 2005 | 02/01 – 03/15 | 8 | 29:13 | 25 | 58 | 1.99 | 11 | 0 | 8 | 0 |
| 2006 | 02/04 – 04/15 | 12 | 67:15 | 70 | 112 | 1.67 | 43 | 1 | 53 | 1 |
| 2007 | 02/01 – 04/29 | 17 | 94:54 | 142 | 188 | 1.98 | 62 | 8 | 54 | 7 |
| 2008 | 02/01 – 04/30 | 17 | 93:33 | 171 | 270 | 2.89 | 57 | 28 | 58 | 34 |
| 2009 | 02/01 – 04/30 | 12 | 68:55 | 82 | 124 | 1.80 | 40 | 10 | 29 | 10 |
| 2010 | 01/25 – 04/30 | 17 | 81:39 | 129 | 182 | 2.23 | 57 | 25 | 39 | 21 |
| 2011 | 02/01 – 05/01 | 15 | 89:59 | 77 | 115 | 1.28 | 28 | 24 | 13 | 20 |
| 2012 – Chiloé | 01/26 – 04/30 | 9 | 47:57 | 12 | 18 | 0.38 | 2 | 4 | 4 | 3 |
| **TOTAL Southern Chile** | | **109** | **591:00** | **710** | **1071** | **1.81** | **304** | **100** | **259** | **96** |
| 2012 – Chañaral | 02/24 – 02/27 | 4 | 26:05 | 17 | 22 | 0.84 | 14 | 0 | 8 | 0 |
| **TOTAL** | | **113** | **617:05** | **727** | **1093** |  | **318** | **100** | **267** | **96** |
