# Supplementary material for: Estimates of Abundance and Trend of Chilean Blue Whales off Isla de Chiloé, Chile
Source: PLoS One. 2017 Jan 12;12(1):e0168646. doi: 10.1371/journal.pone.0168646 (PMC5231374; doi:10.1371/journal.pone.0168646)
Supplement: S2 Table — (DOCX) [file pone.0168646.s003.docx]

**Table S2.** Summary of results from U-CARE tests of goodness-of-fit between the left and right side datasets and various Cormack-Jolly-Seber models. Tests that are significant at p < 0.05 are indicated by an asterisk. With the statistic for trap dependence, positive values indicate ‘trap shyness’ and negative values ‘trap happiness’.

| **Test type** | **Left side** | | **Right side** | |
| --- | --- | --- | --- | --- |
| **3.SR** |  | |  | |
| N(0,1) statistic for transience | 2.23 */* | | 1.59 | |
| Log-odds ratio statistic for transience | 2.00 */* | | 1.52 | |
| χ**^2^** overall | 6.64 | | 4.06 | |
| G2 overall | 6.37 | | 3.94 | |
| By year: | **χ^2^** | **G2** | **χ^2^** | **G2** |
| 2005 | 0.00^A^ | 0.00^A^ | N/A | N/A |
| 2006 | 0.62 | 0.62 | 0.54 | 0.54 |
| 2007 | 0.38 | 0.37 | 0.03 | 0.03 |
| 2008 | 0.31 | 0.31 | 0.90 | 0.88 |
| 2009 | 4.50* | 4.24* | 2.33 | 2.23 |
| 2010 | 0.83 | 0.83 | 0.27 | 0.27 |
| 2011 | 0.00^A^ | 0.00^A^ | 0.00^A^ | 0.00^A^ |
| **3.Sm** |  | |  | |
| χ**^2^** overall | 0.11 | | 2.64 | |
| G2 overall | 0.11 | | 2.73 | |
| By year: | **χ^2^** | **G2** | **χ^2^** | **G2** |
| 2005 | 0.00^A^ | 0.00^A^ | N/A | N/A |
| 2006 | 0.00 | 0.00 | 0.00 | 0.00 |
| 2007 | 0.00 | 0.00 | 0.36 | 0.36 |
| 2008 | 0.11 | 0.11 | 0.63 | 0.64 |
| 2009 | 0.00 | 0.00 | 1.66 | 1.74 |
| 2010 | 0.00^A^ | 0.00^A^ | 0.00^A^ | 0.00^A^ |
| **2.CT** |  | |  | |
| N(0,1) statistic for trap dependence | -0.01 | | -0.49 | |
| Log-odds ratio statistic for trap dependence | -0.40 | | -0.44 | |
| χ**^2^** overall | 0.99 | | 2.01 | |
| G2 overall | 0.99 | | 2.01 | |
| By year: | **χ^2^** | **G2** | **χ^2^** | **G2** |
| 2005 | 0.00 | 0.00 | N/A | N/A |
| 2006 | 0.00 | 0.00 | 0.00 | 0.00 |
| 2007 | 0.13 | 0.13 | 1.43 | 1.43 |
| 2008 | 0.40 | 0.39 | 0.35 | 0.34 |
| 2009 | 0.10 | 0.10 | 0.23 | 0.23 |
| 2010 | 0.36 | 0.36 | 0.00 | 0.00 |
| **2.CL** |  | |  | |
| χ**^2^** overall | 2.30 | | 8.29 | |
| G2 overall | 2.38 | | 8.69 | |
| By year: | **χ^2^** | **G2** | **χ^2^** | **G2** |
| 2005 | 0.00 | 0.00 | N/A | N/A |
| 2006 | 0.27 | 0.27 | 0.00 | 0.00 |
| 2007 | 1.67 | 1.74 | 6.11* | 6.52* |
| 2008 | 0.10 | 0.10 | 0.15 | 0.15 |
| 2009 | 0.27 | 0.27 | 2.02 | 2.02 |
| **Overall** |  |  |  |  |
| χ**^2^** overall | 10.04 |  | 17.01 |  |

^A^ insufficient data for testing
