# Supplementary material for: Estimates of Abundance and Trend of Chilean Blue Whales off Isla de Chiloé, Chile
Source: PLoS One. 2017 Jan 12;12(1):e0168646. doi: 10.1371/journal.pone.0168646 (PMC5231374; doi:10.1371/journal.pone.0168646)
Supplement: S3 Table — (DOCX) [file pone.0168646.s004.docx]

**Table S3**. Chilean blue whale abundance estimates from closed mark-recapture models calculated in CAPTURE. Years of survey are shown in parentheses

| Test | Left side (2004-2012) | | Right side (2005-2012) | |
| --- | --- | --- | --- | --- |
|  | N | Model selection | N | Model selection |
| M(0) | 612 (531-719) | 0.09 | 475 (413-556) | 0.11 |
| M(t) | 585 (510-685) | 0.82 | 455 (398-530) | 0.98 |
| M(b) | N/A | 0.00 | 426 (342-663) | 0.06 |
| M(h) | 849 (736-992) | 0.07 | 636 (544-759) | 0.00 |
| M(h) Chao | 756 (615-961) | 0.07 | 552 (455-696) | 0.00 |
| M(bh) | 306 (304-312) | 0.09 | 259 (258-264) | 0.06 |
| M(th) Chao | **741 (607-937)** | **1.00** | **542 (447-685)** | **1.00** |
| M(tb) | 765 (429-4515) | 0.09 | 362 (293-711) | 0.32 |
| M(t) Chao | 675 (559-844) | 0.82 | 496 (418-614) | 0.98 |
| M(tbh) | N/A | 0.38 | N/A | 0.23 |
