# Supplementary material for: Estimates of Abundance and Trend of Chilean Blue Whales off Isla de Chiloé, Chile
Source: PLoS One. 2017 Jan 12;12(1):e0168646. doi: 10.1371/journal.pone.0168646 (PMC5231374; doi:10.1371/journal.pone.0168646)
Supplement: S4 Table — (DOCX) [file pone.0168646.s005.docx]

**Table S4.** Summary of top p/ϕ-identifiable POPAN models explored in MARK

| Model | # Parameters | AIC | ΔAIC | AIC_c_ weight | Model Likelihood |
| --- | --- | --- | --- | --- | --- |
| Left side (2004-2012) |  |  |  |  |  |
| ϕ(.) p(1=2=9,3=7,t) PENT(1=5,2=8,t) N(.) | 13 | 685.87 | 0.00 | 0.15 | 1.00 |
| ϕ(.) p(1=2=9,3=7,t) PENT(1=5,3=6,t) N(.) | 13 | 685.87 | 0.00 | 0.15 | 1.00 |
| ϕ(.) p(1=2=9,3=7,t) PENT(1=5,3=6,2=8,t) N(.) | 12 | 685.87 | 0.00 | 0.15 | 1.00 |
| ϕ(.) p(1=2=9,3=7,t) PENT(1=5,3=4=6,2=8,t) N(.) | 11 | 685.87 | 0.00 | 0.15 | 1.00 |
| ϕ(.) p(1=2=9,3=7,t) PENT(1=5,3=4=6=7,2=8) N(.) | 10 | 685.87 | 0.00 | 0.15 | 1.00 |
| ϕ(.) p(1=2=9,3=7,t) PENT(1=5,t) N(.) | 14 | 685.87 | 0.00 | 0.15 | 1.00 |
| ϕ(.) p(1=2=9,3=7,t) PENT(t) N(.) | 15 | 687.97 | 2.10 | 0.05 | 0.35 |
| ϕ(.) p(3=8,t) PENT(t) N(.) | 17 | 689.08 | 3.21 | 0.03 | 0.20 |
| ϕ(.) p(1=2=9,t) PENT(t) N(.) | 16 | 689.69 | 3.83 | 0.02 | 0.15 |
| ϕ(.) p(t) PENT(t) N(.) | 18 | 691.18 | 5.31 | 0.01 | 0.07 |
| ϕ(.) p(1=2,t) PENT(t) N(.) | 17 | 691.19 | 5.33 | 0.01 | 0.07 |
| Right side (2005-2012) |  |  |  |  |  |
| ϕ(.) p(1=8,2=6,5=7,t) PENT(t) N(.) | 12 | 632.21 | 0 | 0.26 | 1.00 |
| ϕ(.) p(1=8,2=6,5=7,t) PENT(6=7,t) N(.) | 11 | 632.21 | 0 | 0.26 | 1.00 |
| ϕ(.) p(1=8,2=6,5=7,t) PENT(3=6=7,t) N(.) | 10 | 632.21 | 0 | 0.26 | 1.00 |
| ϕ(.) p(1=8,2=6,t) PENT(t) N(.) | 13 | 634.30 | 2.09 | 0.09 | 0.35 |
| ϕ(.) p(1=8,2=6,5=7,t) PENT(1=2,t) N(.) | 11 | 634.31 | 2.11 | 0.09 | 0.35 |
| ϕ(.) p(1=8,t) PENT(t) N(.) | 14 | 636.38 | 4.17 | 0.03 | 0.12 |
| ϕ(.) p(t) PENT(t) N(.) | 16 | 638.45 | 6.24 | 0.01 | 0.04 |
| ϕ(.) p(1=2,t) PENT(t) N(.) | 15 | 638.46 | 6.25 | 0.01 | 0.04 |
