# Supplementary material for: Estimates of Abundance and Trend of Chilean Blue Whales off Isla de Chiloé, Chile
Source: PLoS One. 2017 Jan 12;12(1):e0168646. doi: 10.1371/journal.pone.0168646 (PMC5231374; doi:10.1371/journal.pone.0168646)
Supplement: S5 Table — (DOCX) [file pone.0168646.s006.docx]

**Table S5.** Summary of top identifiable Pradel models explored in MARK

| Model | # Parameters | AIC | ΔAIC | AIC_c_ weight | Model Likelihood |
| --- | --- | --- | --- | --- | --- |
| Left side (2004-2012) |  |  |  |  |  |
| ϕ(.) p(1=2=9,3=6,4=7,t) λ(1=5,3=6=7=8,t) | 10 | 1811.31 | 0.00 | 0.33 | 1.00 |
| ϕ(.) p(3=6,4=7,t) λ(3=6=7=8,t) | 13 | 1813.21 | 1.90 | 0.13 | 0.39 |
| ϕ(.) p(1=9,3=6,4=7,t) λ(3=6=7=8,t) | 12 | 1813.21 | 1.90 | 0.13 | 0.39 |
| ϕ(.) p(3=6,4=7,t) λ(1=5,3=6=7=8,t) | 12 | 1813.21 | 1.90 | 0.13 | 0.39 |
| ϕ(.) p(1=2=9,3=6,4=7,t) λ(3=6=7=8,t) | 11 | 1813.34 | 2.03 | 0.12 | 0.36 |
| ϕ(.) p(3=6,4=7,t) λ(3=6=8,t) | 14 | 1815.26 | 3.95 | 0.05 | 0.14 |
| ϕ(.) p(3=6,4=7,t) λ(1=2,3=6=8,t) | 13 | 1815.26 | 3.95 | 0.05 | 0.14 |
| ϕ(.) p(3=6,4=7,t) λ(2=4,3=6=8,t) | 13 | 1815.34 | 4.03 | 0.04 | 0.13 |
| ϕ(.) p(3=6,4=7,t) λ(3=8,t) | 15 | 1817.36 | 6.05 | 0.02 | 0.05 |
| ϕ(.)p(1=2=9,3=6,4=7,t) λ(.)* | 7 | 1821.54 | 10.23 | 0.00 | 0.01 |
| Right side (2005-2012) |  |  |  |  |  |
| ϕ(.) p(1=8,2=3=6=7,t) λ(1=5,3=6,2=7,t) | 9 | 1540.72 | 0.00 | 0.41 | 1.00 |
| ϕ(.) p(1=8,2=3=6=7,t) λ(1=5,3=6,2=4=7,t) | 8 | 1541.86 | 1.13 | 0.23 | 0.57 |
| ϕ(.) p(1=8,2=3=6=7,t) λ(1=5,3=6,t) | 10 | 1542.15 | 1.43 | 0.20 | 0.49 |
| ϕ(.) p(1=8,2=3=6,t) λ(1=5,3=6,t) | 11 | 1543.94 | 3.22 | 0.08 | 0.20 |
| ϕ(.) p(1=8,2=3=6,t) λ(1=5,t) | 12 | 1545.64 | 4.92 | 0.04 | 0.09 |
| ϕ(.) p(1=8,2=3,t) λ(1=5,t) | 13 | 1547.79 | 7.07 | 0.01 | 0.03 |
| ϕ(.) p(1=8,2=3=6,t) λ(.)* | 7 | 1549.40 | 8.68 | 0.01 | 0.01 |

*models shown for AICc weight>0.01, with best AICc time-invariant λ model also shown
